# ANO4 (Anoctamin 4) Is a Novel Marker of Zona Glomerulosa That Regulates Stimulated Aldosterone Secretion

**DOI:** 10.1161/HYPERTENSIONAHA.119.13287

**Published:** 2019-09-30

**Authors:** Carmela Maniero, Paolo Scudieri, Lalarukh Haris Shaikh, Wanfeng Zhao, Mark Gurnell, Luis J.V. Galietta, Morris J. Brown

**Affiliations:** 1 From the Centre for Clinical Pharmacology, William Harvey Research Institute, Barts and the London School of Medicine & Dentistry, Queen Mary University of London, United Kingdom Clinical Pharmacology Unit (C.M., L.H.S., M.J.B.); 2 Telethon Institute of Genetics and Medicine (TIGEM), Pozzuoli, Italy (P.S., L.J.V.G.); 3 Human Research Tissue Bank, Cambridge University, Hospitals NHS Foundation Trust, Addenbrooke’s Hospital, United Kingdom (W.Z.); 4 Metabolic Research Laboratories-Wellcome Trust-MRC Institute of Metabolic Science (M.G.).

**Keywords:** aldosterone, anoctamins, cell proliferation, chloride channel, Zona glomerulosa

## Abstract

Microarray comparison of the transcriptomes of human adrenal zona glomerulosa (ZG) and zona fasciculata found several ZG-specific genes that negatively regulate aldosterone secretion. The third and most significantly upregulated ZG-gene (19.9-fold compared with zona fasciculata, *P*=6.58×10^−24^) was *ANO4*, a putative Ca^2+^-activated chloride channel. We have investigated the role of *ANO4* in human adrenal, and whether it functions like the prototype anoctamin, *ANO1*. We evaluated *ANO4* mRNA and protein expression in human adrenal by qPCR and immunohistochemistry, compared the effects of *ANO4* and *ANO1* overexpression on baseline and stimulated aldosterone secretion and cell proliferation in H295R cells, and analyzed ANO4 activity as a Ca^2+^-activated chloride channel in comparison with other anoctamins by a fluorescence-based functional assay. The expression of *ANO4* in ZG was confirmed by qPCR as 23.21-fold upregulated compared with zona fasciculata (n=18; *P*=4.93×10^−7^). Immunohistochemistry found cytoplasmic, ZG-selective expression of ANO4 (anoctamin 4) protein. *ANO4* overexpression in H295R cells attenuated calcium-mediated aldosterone secretion and cell proliferation in comparison to controls. The latter effects were in a different direction to those of ANO1. The functional assay showed that, in contrast to ANO1, ANO4 expression results in low levels of calcium-dependent anion transport. In conclusion, ANO4 is one of the most highly expressed genes in ZG. It attenuates stimulated aldosterone secretion and cell proliferation. Although belonging to a family of Ca^2+^-activated chloride channels, it does not generate significant plasma membrane chloride channel activity.

## Abstract 2

Supplemental Digital Content is available in the text.

Although zona glomerulosa (ZG) is the main physiological site for aldosterone production, in the adult adrenal gland aldosterone synthase expression is suppressed in the majority of the ZG cells and confined to the aldosterone-producing cell clusters,^1^ which carry somatic mutations in the same genes (*ATP1A1, ATP2B3, CACNA1D*) that induce constitutive *CYP11B2* expression and aldosterone production in aldosterone producing adenomas (APAs).^2–4^

We have previously undertaken a microarray study comparing the transcriptome of adjacent ZG and zona fasciculata (ZF) of 21 adrenals. Most of the highly selective ZG markers studied so far (*LGR5, DACH1, NEFM*) negatively regulate aldosterone production and cellular proliferation.^5–7^ The third most ZG-selective gene (19.9-fold more expressed in ZG compared with ZF), and most significantly upregulated (*P*=6.58×10^−24^), was *ANO4*, a member of the anoctamin family, which includes 10 paralogues (ANO1-10) playing different physiological functions and involved in several diseases.^8^ ANO1 and ANO2 are well-characterized calcium-activated chloride channels (CaCCs).^9–11^ ANO1 is involved in fluid secretion in secretory epithelia and eccrine glands, smooth muscle contraction, tumorigenesis, and cell proliferation.^8,9^ ANO2 is expressed in the nervous system where it mediates olfaction and synaptic transmission.^12,13^ Anoctamins also regulate intracellular Ca^2+^ signaling in different subcellular compartments, from endoplasmic reticulum (ER) stores to lipid raft signaling platforms.^14^

ANO4 (anoctamin 4) function is debated: it has been described as CaCC in Human Embryonic Kidney (HEK)293-transfected cells^15^ but also as a phospholipid scramblase involved in apoptosis^16^ or even as a non-selective cation channel.^17^ Another recent article reported that ANO4 colocalizes with the sarcoendoplasmic reticulum Ca^2+^-ATPase and reduces Ca^2+^ store release, probably acting as a leakage channel.^18^

Until recently, most or all of the evidence concerning membrane polarization and ion traffic in the regulation of aldosterone secretion has pointed to cation channels and transporters. The discovery that the chloride channel, *CLCN2*, has germline mutations, which increase excitatory efflux of chloride ions, in families with early onset of PA^19,20^ creates considerable interest in the role of another putative chloride channel whose expression shows a high degree of ZG selectivity. We have investigated the role of ANO4 in regulating human ZG function, using measures of aldosterone secretion and cell proliferation. We have studied ANO4’s influence on responses to Angiotensin II, ionomycin, and ATP, which increase intracellular calcium by different mechanisms (IP_3_-stimulated ER calcium release and voltage-dependent T- and L-type calcium channels, release from ER stores and via purinergic receptor activation, respectively)^21–23^ and for comparison have studied ANO1, a well-characterized, ubiquitous CaCC.

## Methods

The data that support the findings of this study are available from the corresponding author on reasonable request.

Further details are provided in the online-only Data Supplement.

### Chemicals

Angiotensin II, ionomycin, and ATP were purchased from Sigma-Aldrich.

### Human Subjects

Human adrenal tissues from patients who underwent adrenalectomy after being diagnosed with unilateral APA or phaeocromocytoma were obtained from Cambridge University Hospitals’ Human Research Tissue Bank post-surgery at Addenbrooke’s Hospital, Cambridge, United Kingdom. All tissues were obtained with approval from the Cambridgeshire Research Ethics Committee with written informed consent before surgery. APAs and their paired adjacent normal adrenal were identified and macroscopically dissected by histopathologists and separated into 3 categories for processing (1) snap-frozen in liquid nitrogen and then stored at −70°C for immunohistochemistry or DNA extraction or (2) stored in RNA-later for RNA extraction or (3) digested with collagenase for 2 hours and then placed in DMEM at 37°C in 5% CO_2_ for cell culture.

Laser capture microdissection, RNA extraction, and microarray analysis were performed as described previously^24^ in the online-only Data Supplement.

### Cell Culture Experimentation

H295R human adrenocortical carcinoma cells were cultured in DMEM/Nutrient F-12 Ham supplemented with 10% calf bovine serum, 100 U penicillin, 0.1 mg/mL streptomycin, 0.4 mmol/L l-glutamine, and insulin-transferrin-sodium selenite medium at 37°C in 5% CO_2_ as previously described.^5^ Gene overexpression was performed using lipid-mediated cell transfection Lipofectamine 3000 (Thermo Fisher). Cells were transfected with GFP-tagged ANO4 (EX-Mm36875-M29, EX-W1648-M29, EX-I1781-M29, EX-T8891-M29, Genecopoeia), or empty vector control. At 24 hours, H295R cells were serum deprived in unsupplemented medium for 12 hours, and further drug treatments were carried out with 24 hours incubation from this point.

Gene silencing was achieved using DharmaFECT 1 lipid transfection reagent (Dharmacon). Cells were silenced using either ON-TARGETplus nontargeting small interfering RNA as control or SMART pool: ON-TARGET plus ANO4 small interfering RNA (Dharmacon; 50 nmol/L). Cells were harvested for analysis of mRNA and protein expression after 48 hours.

### qPCR Analysis of Gene Expression

Cells were kept in TRIzol (Ambion). Total DNA-free RNA was isolated using the PureLink RNA minikit with the PureLink deoxyribonuclease set (Life Technologies) according to manufacturer’s instructions, with an in-column DNAse step (Qiagen). Reverse transcription was performed using the reverse transcriptase system (Promega) with a 1:1 mixture of random and oligo primers according to the manufacturer’s instructions. mRNA expression of genes of interest was quantified using TaqMan probes (Applied Biosystems), and CYP11B2 expression was quantified using custom-made probes as specified before.^15^ The housekeeping 18S rRNA (Applied Biosystems) was used for normalization. Reverse transcription was performed using the reverse transcriptase system (Promega) with a 1:1 mixture of random and oligo primers according to the manufacturer’s instructions. mRNA expression of genes of interest was quantified using TaqMan probes (Applied Biosystems), and *CYP11B2* expression was quantified using custom-made probes as specified before.^25^

### Aldosterone and Protein Measurement

Commercially available Homogenous Time Resolved Fluorescence Resonance Energy Transfer assay from Cisbio Bioassays, France was used according to manufacturer’s instructions. The aldosterone concentrations from H295R cells were normalized to total cell protein, which was determined by performing the bicinchoninic acid protein assay (Pierce Biotechnology).

### Western Blotting

Western blotting was conducted using total protein extracts. Proteins were separated by electrophoresis in 8% to 10% SDS-polyacrylamide gel and transferred to a polyvinylidene fluoride membrane, immunoblotted with anti-NEFM (Sigma-Aldrich, United Kingdom; 1:500 dilution). Anti-Glyceraldehyde-3-Phosphate Dehydrogenase (GAPDH) (#G8795, Sigma, United Kingdom; 1:10 000 dilution) was used as an internal control for protein abundance. HRP-conjugated ssecondary anti-rabbit or anti-mouse antibodies were used. The bands were detected using the Pierce ECL Western Blotting Substrate (Thermo Fisher Scientific).

### Halide-Sensitive Yellow Fluorescent Protein Assay

The HS-YFP (halide-sensitive yellow fluorescent protein) assay is a cell-based test which utilizes an engineered halide-sensitive yellow fluorescent protein.^26^ This assay detects the iodide (I^−^) influx as a reporter of anion channel activity. Cells expressing HS-YFP and anoctamins are exposed to an I^−^-rich solution and a calcium agonist such as Ionomycin at different concentrations. CaCC function is calculated from the rate of cell fluorescence quenching in response to extracellular addition of I^−^ (100 mmol/L) plus ionomycin. In the case of CaCCs, activation by ionomycin, or other calcium agonists, results in accelerated HS-YFP quenching because of CaCC-mediated I^−^ entry.

HEK-293 cells were seeded in 96-well microplates (25 000 cells/well) in 100 µL of antibiotic-free culture medium. After 6 hours, cells were cotransfected with plasmids carrying the coding sequence for anoctamin constructs and the HS-YFP. For each well, 0.2 µg of total plasmid DNA and 0.5 µL of lipofectamine 2000 were first premixed in 60 µL optimem to generate transfection complexes (60 minutes at RT) and then added to the cells. After 24 hours, the fresh culture medium plus antibiotics replaced transfection media. The HS-YFP functional assay was performed 48 hours after transfection. Cells were washed 2× with PBS and incubated for 20 minutes with 60 μL of PBS. After incubation, cells were transferred to a microplates reader (FluoStar Galaxy; BMG Labtech, Ortenberg, Germany) for CaCC activity determination. The plate reader was equipped with high-quality excitation (ET500/20×) and emission (ET535/30 m) filters for YFP (Chroma Technology Corp, Brattleboro, VT). Each assay consisted of a continuous 14 s fluorescence reading with 2 s before and 12 s after injection of 165 μL of modified PBS (Cl^−^ replaced by l^−^ with final concentration 100 mmol/L) also containing 1 or 10 μM ionomycin. Data were normalized to the initial, background-subtracted, fluorescence. To determine fluorescence quenching rate associated with l^−^ influx, the final 11 seconds of the data for each well were fitted with an exponential function to extrapolate the initial slope (dF/dt).

### XTT Assay

Cells were grown in a 96-well plate at a density of 10^4^ cells/well in 100 µL of culture medium and transfected with Lipofectamine. After 48 hours from transfection, the assay was performed: 25 µL of XTT/PMS (N-methyl dibenzopyrazine methyl sulfate) solution were added directly to each well containing 100 µL cell culture. The solution was made as described: 1 mg of XTT was dissolved in 1 mL of warm culture media. Ten microliters of the PMS solution (10 mmol/L PMS solution in PBS) were added to the 4 mL. Cells were incubated for 2 hours at 37°C in a CO_2_ incubator. The plate was read at 450 nm in a plate reader.

### Immunohistochemistry

Immunohistochemistry was performed on formalin-fixed, paraffin-embedded sections (4 μm) using an automated immunostainer with cover tile technology (Bond-III system, Leica Biosystems). The commercial antibody anti-ANO4 (Sigma, HPA053412) and anti-CYP11B2 antibody (a kind gift from Dr Celso E. Gomez-Sanchez) were used as the primary antibodies. The antigen retrieval was carried out using the combination of heat and Bond Epitope Retrieval Solution 1 (Leica, AR9961) for 20 minutes. The optimal working dilution for ANO4 was 1/50. The Bond Polymer Refine Detection kit (Leica, DS9800) was used for detecting and visualizing the antigens. Negative controls, in which primary antibodies were omitted, resulted in a complete absence of staining.

### Immunofluorescence

H295R cells were cultured on sterilized and poly l-lysine coated cover slips for 24 hours. After lipofectamine transfection, cell plasma membranes were stained with Wheat Germ Agglutinin, Alexa Fluor 633 Conjugate (W21404, Life Technologies) for 10 minutes at 37°C. Cells were washed twice with PBS (5 minute each), fixed with 4% paraformaldehyde and permeabilized with 1% trition-X100 in PBS, 10 minutes each at room temperature. Finally, cells were washed 3× in PBS, and cover slips were mounted on slides using Vectashield Antifade Mounting Medium with DAPI (H-1200, Vector Laboratories). Confocal images were taken using Zeiss LSM510 Meta confocal microscope and analyzed using Zen 2011 software.

### Data Analysis

Each experiment was performed with biological replicates, and the averages were calculated. Results are expressed as mean values with SEM. Differences between 2 groups were analyzed for statistical significance by *t* test, and multiple groups were analyzed by 1- or 2-way ANOVA followed by Tukey post hoc test. The significance level of *P*<0.05 was considered to indicate statistical significance. Statistical analysis was performed as indicated using the standard statistical software, Prism 6 (GraphPad Software, Inc) and GENMOD procedure of SAS.

## Results

### ANO4 Expression in Zona Glomerulosa and APAs

**Figure 1.**
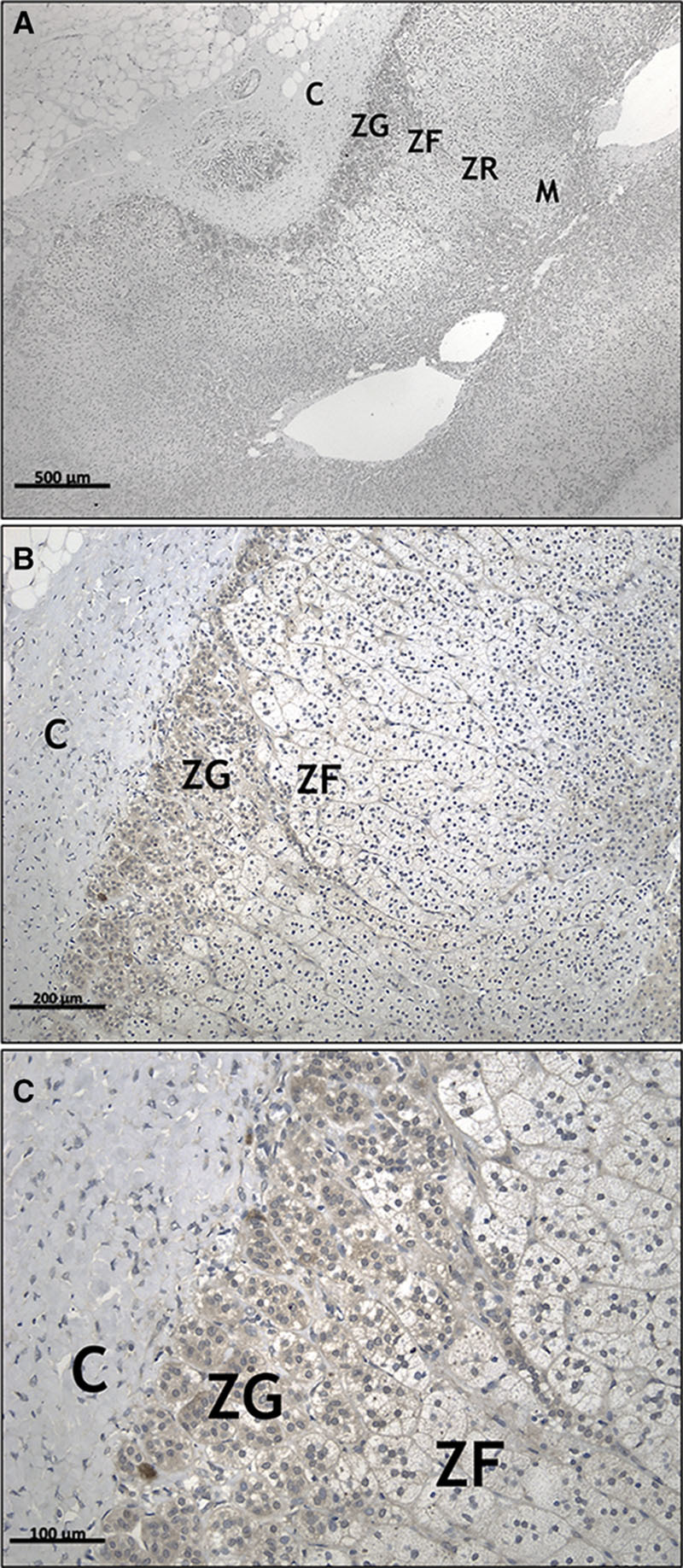
Immunohistochemistry of ANO4 (anoctamin 4) in formalin-fixed paraffin-embedded human normal adrenal cortex. ANO4 is selectively expressed in zona glomerulosa (ZG) cells. The staining shows a diffuse cytoplasmic pattern. **A**–**C**, Shows 4×, 10×, and 20× magnification, respectively. These images are representative of 6 observations. C indicates capsule; M, medulla; and ZF, zona fasciculata.

The expression of ANO4 in ZG was confirmed by qPCR as 23.21-fold upregulated compared with ZF (n=18; *P*=4.93×10^−7^). Its expression in APAs was similar to ZF, and there were no differences between KCNJ5 mutant and wild-type adenoma (Figure S1 in the online-only Data Supplement). ANO4 protein expression in the normal adrenal glands adjacent to APA or phaeocromocytoma was highly selective for ZG versus ZF. It was also detected in the medulla. The staining pattern was mainly cytoplasmic (Figure 1).

There was no correlation between the distribution of ANO4 protein expression, homogeneous in the ZG, and CYP11B2, which was typically patchy (Figure S2).

### ANO4 and ANO1 Modulate Aldosterone Secretion in Different Directions

**Figure 2.**
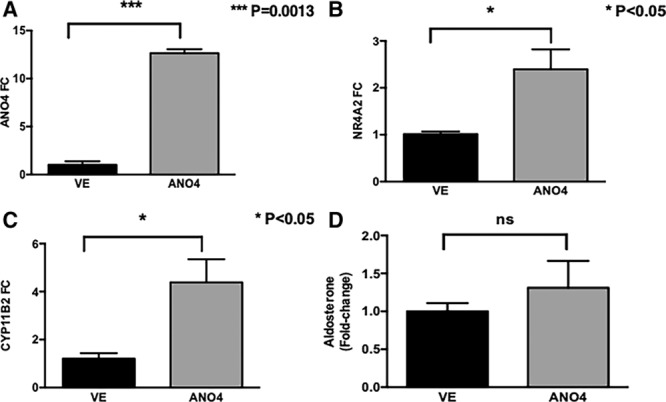
Effect of *ANO4* overexpression on basal aldosterone production. Overexpression of *ANO4* in H295R cells increased *ANO4* mRNA by 12.6-fold (**A**), and increased *NR4A2* (**B**), and *CYP11B2* (**C**) mRNA levels but did not affect aldosterone production (**D**) in comparison with control (EV=empty vector). Similarly, silencing *ANO4* for 48 h with small interfering RNA significantly reduced ANO4 (anoctamin 4) protein expression (**C**) but did not affect aldosterone secretion (**D**). Data are shown in geometric mean values ±SEM and are representative of 4 experiments.

Overexpression of *ANO4* in H295R cells increased *ANO4* mRNA by 12.6 fold in transfected cells compared with the empty vector (*P*<0.001; Figure 2A). GFP fluorescence pattern in transfected cells was mainly cytoplasmic (Figure S3). Although this resulted in an increase in NR4A2 and CYP11B2 expression (Figure 2B and 2C), basal aldosterone secretion was not affected (Figure 2C).

**Figure 3.**
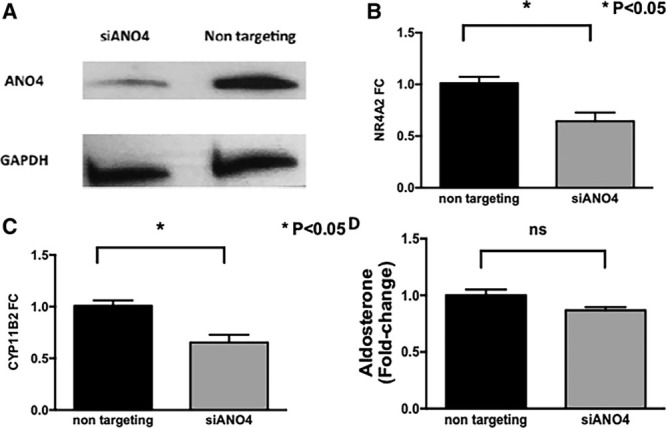
Effect of *ANO4* knockdown on basal aldosterone production. Silencing *ANO4* for 48 h with small interfering RNA significantly reduced ANO4 (anoctamin 4) protein expression (**A**) and *NR4A2* (**B**), and *CYP11B2* (**C**) mRNA levels but did not affect aldosterone secretion (**D**). Data are shown in geometric mean values ±SEM and are representative of 4 experiments.

Similarly, silencing of ANO4 protein (Figure 3A), despite a downregulation of NR4A2 and CYP11B2 (Figure 3B and 3C), did not cause any significant change in aldosterone secretion in comparison to control (Figure 3D).

**Figure 4.**
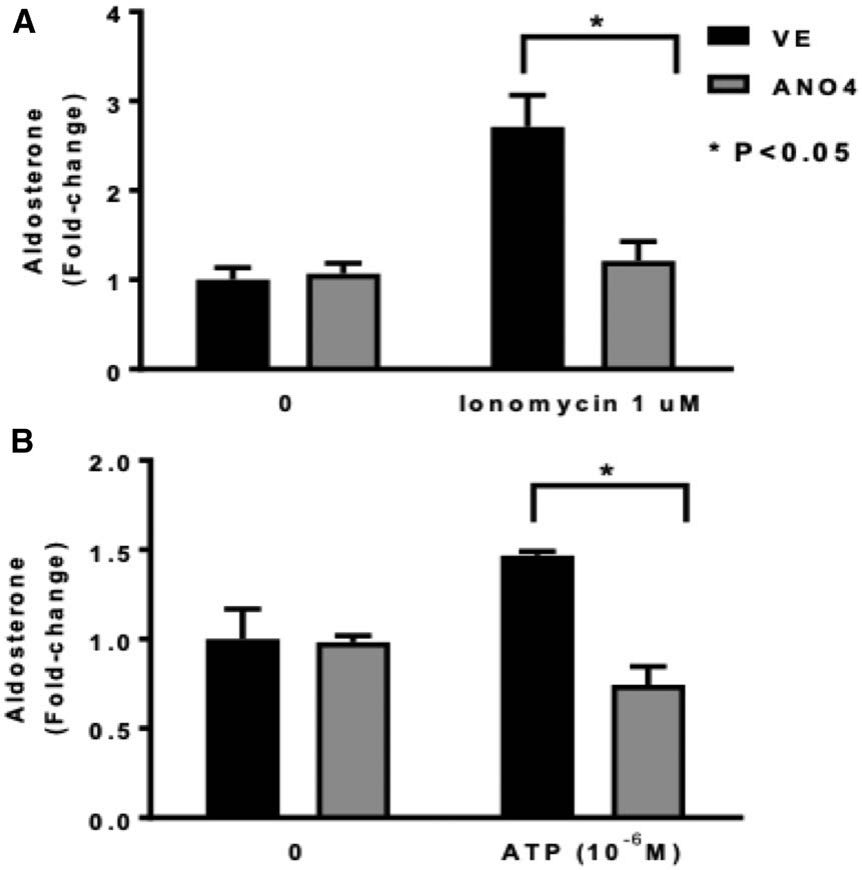
Effect of ANO4 (anoctamin 4) overexpression in H295R on ionomycin (**A**) and ATP-stimulated aldosterone secretion. Ionomycin increased aldosterone secretion by 2.7-fold (vs basal conditions) in controls, and only 1.2-fold in ANO4-transfected cells (*P<0.05, **A**). ATP increased aldosterone secretion by 1.5-fold (vs basal aldosterone) in controls, while it had no effect in ANO4-transfected cells (*P<0.05), **B**). Data are shown in geometric mean values+SEM.

Ionomycin, which causes an increase of intracellular Ca^2+^ via release from the ER stores,^21^ increased aldosterone by 2.7-fold in controls, whereas in ANO4-transfected cells aldosterone secretion increased by 1.2 fold (*P*<0.05 versus stimulated controls; Figure 4A). Similarly, ATP, which increases intracellular Ca^2+^ via purinergic receptors^22^ showed similar trends (*P*<0.05 versus stimulated controls; Figure 4B).

Of note, the aldosterone secretagogue effect of AngII (10^−8^ M), which also increases intracellular Ca^2+^ concentration^23^ and as expected caused a significant stimulation in aldosterone production in cells transfected with empty vector, was attenuated, although not significantly, by ANO4 transfection (Figure S4A).

In contrast to ANO4, ANO1 overexpression in H295R cells enhanced basal aldosterone production by 2-fold (*P*<0.05 versus controls; Figure S4A).

### ANO4 and ANO1 Regulate Cell Proliferation

Basal proliferation of cells transfected with ANO1 and ANO4 was 1.8- and 1.6-fold higher than controls, respectively, as detected by XTT assay (*P*<0.05; Figure S4B). In presence of the calcium-elevating agent ionomycin (1 µM), proliferation was further stimulated only in cells overexpressing ANO1 by 2.3-fold, whereas in ANO4-transfected cells, no difference was observed in comparison to basal conditions (*P*<0.05; Figure S4B).

### ANO4 Is Not a CaCC in HEK293 Cells

**Figure 5.**
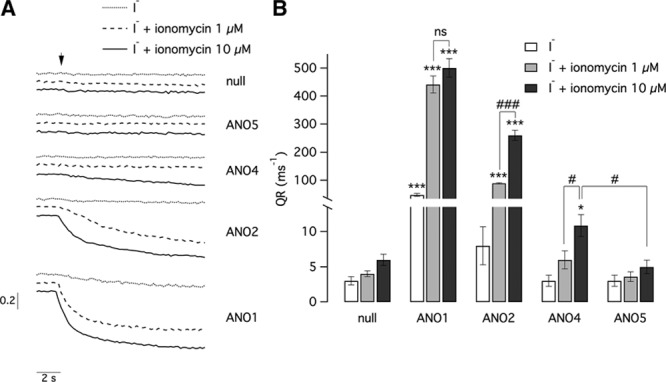
Representative traces (left) and summary of data (right) from experiments carried out with the HS-YFP (halide-sensitive yellow fluorescent protein) assay on HEK-293 cells transiently transfected with ANO1, ANO2, ANO4 (anoctamin 4), and ANO5 or empty plasmids (null). Traces show the cell fluorescence decrease following I^−^ addition (arrow) with or without ionomycin (1 or 10 µmol/L). The bar graph reports the maximal quenching rate (QR) for the indicated conditions. Data are from 6 to 13 experiments. Asterisks (*P<0.05; ***P<0.001) indicate a statistically significant difference vs cells transfected with empty vectors. Hash signs indicate a statistically significant difference between indicated groups of data (#*P*<0.05; ###*P*<0.001).

As expected, intracellular calcium elevation triggered by ionomycin (1 and 10 µmol/L) caused a large flow of iodide in cells expressing ANO1 and ANO2. A higher response to ionomycin 10 versus 1 µmol/L was found for ANO2 but not with ANO1. ANO4 expression was associated with a much lower rate of anion transport. Even with the highest ionomycin concentration, the quenching rate in ANO4 cells was >40-fold and 20-fold lower than that of ANO1 and ANO2 cells, respectively. However, despite this low value, anion transport in ANO4 cells was significantly higher than that of ANO5, which is an anoctamin with prevalent intracellular localization^27^ (*P*<0.05 versus ANO1; Figure 5).

## Discussion

The comparison of ZG versus ZF transcriptomes has found several unsuspected genes upregulated in human ZG. *ANO4* was numerically the third most upregulated gene in ZG and statistically the most significant.

Our previous functional studies of 3 of the ZG-specific genes, *DACH1*,^5^ *LGR5*,^6^ and *NEFM*^7^ showed that they suppress aldosterone secretion and proliferation.

These results presented here show that, while *ANO4* does not affect basal aldosterone secretion, it suppresses cell proliferation and aldosterone secretion in conditions of increased intracellular calcium levels. It is possible that, while net aldosterone secretion is unchanged, this is because an initial change in CYP11B2 activity (for instance, in response to Ca^2^^+^-mediated activation of the STAR protein) causes compensatory changes of CYP11B2 transcription in the opposite direction, likely driven by NR4A2.^28, 29^

Our hypothesis is that most ZG-selective transcripts found in our microarray analysis are negative regulators of aldosterone production. Some ZG-selective genes have their effect mainly on basal aldosterone production (*DACH1*, *LGR5*) while others dampen or inhibit the aldosterone response to stimuli (*ANO4*, *NEFM*). These observations are consistent with the finding that in human ZG there is little expression of CYP11B2 except in aldosterone-producing cell clusters.

ANO4 belongs to the anoctamin family, which includes CaCCs such as ANO1 and ANO2. CaCCs conducted currents have been observed in almost all tissues, with different physiological functions. However, inconsistent data have been published about ANO3-10 members. Duran et al^27^ described ANO3-7 as intracellular proteins, which did show features of plasma membrane CaCCs. Tian et al^15^ reported that, when co-expressed with the purinergic receptor P2Y2 and in presence of ATP, ANO4 induces hyperpolarization of plasma cell membrane, while most of the other anoctamins caused depolarization. In other studies, anoctamins other than ANO1 and ANO2 have been found to function as phospholipid scramblases rather than as ion channels.^16^

Our YFP assay results showed that, while ANO1 and ANO2 generated large flows of iodide in a pattern consistent with the different calcium sensitivity of the 2 proteins,^30^ ANO4 expression was associated with a significantly lower rate of anion transport, although slightly higher than ANO5, that has prevalent intracellular localization,^27^ therefore ruling out a role as CaCC.

More recently, ANO4 and ANO1 were found to affect compartmentalized calcium signals and stores in opposite directions: while the latter is localized in plasma membrane rafts, facilitates local Ca^2+^ release from the ER, and is activated by store release Ca^2+^, ANO4, which may also be permeable to calcium,^15^ is localized on the ER membrane, is activated by SOCE, lowers the Ca^2+^ store contents, and mediates Ca^2+^ leakage out of the ER.^18^ Moreover, cytosolic Ca^2+^ increase by ATP or ionomycin-induced ER-store release was reduced in ANO4-expressing cells.^21^ We speculate that this is a possible mechanism responsible for the functional effects on aldosterone production observed in our in vitro experiments.

Extensive data from work on adrenocortical cells,^23^ and of animal models of PA because of deletion of ion channels controlling adrenocortical membrane polarization^31,32^ pointed to the likely importance of intracellular calcium regulation by membrane polarization. This prediction has been largely confirmed by the finding of gain-of-function somatic mutations affecting cation transport in most aldosterone-producing adenomas, and in particular the finding of multiple mutations affecting calcium influx in the gene, CACNA1D, encoding Cav1.3. Another membrane Ca^2+^-sensor, VSNL1, is selectively expressed in ZG and APAs.^33^ Until recently, such studies pointed to cation channels and transporters as the main regulators of aldosterone secretion. But the recent discovery of germline mutations in the chloride channel, CLCN2, in rare families with early onset PA illustrates the potential importance also of anion channels.^19,20^

To establish the relationship between anoctamin expression and other channels already known to be active in the adrenal gland, we looked into published articles. Chloride currents recorded in the adrenal of human and nonhuman species have different characteristics from CaCCs: Chorvatova et al^34^ described a Ras-dependent, transient chloride current activated by ACTH at low concentrations in bovine ZG cells through activation of Ras by β gamma subunits.^35^ Spat et al examined Cl^−^ currents with the patch-clamp technique in rat ZG cells. With the application of nearly symmetrical Cl^−^ concentration, and after inhibiting K^+^ currents, they did not find any significant channel activity. However, in a significant fraction of the cells, the slow activation of a tiny inward current could be observed at strongly negative voltages. This was significantly accentuated by reducing extracellular pH. Both the kinetic and pharmacological characteristics of this inwardly rectifying current suggest that it passes through chloride ClC-2 channels.^23,36^ Therefore, there is no evidence of channels resembling CaCCs.

One limitation of our study is the fact that as normal adrenals are rarely removed, we have used those adjacent to either an APA or pheochromocytoma, the most common indications for adrenalectomy in our hospital. However, there was no difference in the expression of ANO4 between these 2 groups of patients. Moreover, ANO4 expression results to seen to be ZG-selective in a published microarray analysis of adrenal glands obtained from kidney donors.^1^ It seems therefore unlikely that its abundance is because of the tumors or to their different effect on sodium balance.

Other limitations are the fact that our proliferation assay was not corrected by transfection rate and that the YFP assay, a well-established high-throughput method evaluating I-fluxes, uses HEK293 cells, while ANO4 expression is selective for aldosterone-secreting, adrenal ZG cells. It is unlikely but still possible that ANO4 has different function in adrenal cells.

In summary, our study showed that *ANO4*, similarly to our previously described ZG-selective upregulated genes (*NEFM, LGR5*, and *DACH1*), inhibits rather than stimulates aldosterone secretion in conditions of intracellular calcium increase. Its electrophysiological properties at high-throughput screening YFP assay do not resemble those of CaCCs. The elucidation of the role played by ANO4 in aldosterone production will be facilitated by a clear understanding of its function as a channel and as a lipid scramblase. The much lower anion transport in ANO4-transfected cells, compared with those expressing ANO1 and ANO2, is probably because of the protein being localized mainly in intracellular compartments. Despite this localization, we could measure a small but significant level of anion transport compared with ANO5. This could indicate that a small amount of ANO4 protein reaches the cell surface.

In this article, we have concentrated on elucidation of the physiological role of *ANO4* in adrenal cortical cells. Our microarray data showed its expression to be much lower, but not absent, in APAs of all genotypes, and microarray data for aldosterone-producing cell clusters shows similar expression of ANO4 in these as in adjacent ZG cells.^1,5,6^ Future studies might investigate localization and role of ANO4 in cells with autonomous aldosterone production. A cell-surface location, and possible role in apoptosis, may open interesting possibilities for ANO4 as a therapeutic target.

## Perspectives

Primary aldosteronism is the most common cause of secondary hypertension, 30% to 50% of cases being represented by APA. 2 APA subtypes have been described, carrying different somatic mutations in ion channels (KCNJ5, ATP2B3, ATP1A1, and CACNA1D) and presenting with different histological phenotypes. Transcriptome analysis found genes upregulated in ZG compared with ZF that inhibit hormone production. Among these, ANO4 inhibits aldosterone production in presence of high intracellular calcium levels. We speculate that the inhibition contributes to the well-documented patchiness of aldosterone production in human ZG, which is attributed to salt excess.

## Sources of Funding

The work was funded by a National Institute for Health Research (NIHR) Senior Investigator Award (NF-SI-0512-10052) to M.J. Brown, and by the NIHR Barts Hospital Biomedical Research Centre. C. Maniero was supported by a British Heart Foundation clinical research fellowship Grant (FS/14/12/30540). L.H. Shaikh was supported by a British Heart Foundation PhD scholarship (FS/11/35/28871; J). M. Gurnell was funded by NIHR Cambridge Biomedical Research Centre (Metabolic).

## Disclosures

None.

## Supplementary Material

## Novelty and Significance

### What Is New?

- We have reported several genes upregulated in zona glomerulosa (ZG) compared with zona fasciculata. Among these, *ANO4* is the third and most significantly ZG-selective transcript.
- *ANO4* is a member of the anoctamin family, which includes 10 paralogues (ANO1-10) playing different physiological functions and involved in several diseases. In particular, *ANO1* is a well-characterized CaCC.
- We have found that *ANO4* plays a functional role in adrenal cells. It attenuates aldosterone secretion and cell proliferation in response to elevated intracellular calcium levels. On the contrary, *ANO1*, a known calcium-activated chloride channel, amplifies aldosterone response to elevated intracellular calcium levels.
- In adrenal zona glomerulosa and in transfected cells, ANO4 (anoctamin 4) localization is mainly cytoplasmic. Compared with other anoctamins, it does not generate calcium-activated chloride currents.

### What Is Relevant?

- All ZG-selective genes investigated so far, including *ANO4*, downregulate aldosterone production and cell proliferation, through different molecular pathways and signaling. Because of the high selectivity of its expression in ZG, ANO4 can be considered a novel potential pharmacological target for new drugs that may modulate aldosterone secretion.

### Summary

The comparison of ZG versus zona fasciculata transcriptomes has revealed several unsuspected genes many-fold upregulated in human ZG. ANO4 was the third most upregulated gene in ZG. These genes suppress rather than stimulate aldosterone secretion, and this finding could be interpreted as an adaptive mechanism to high-salt intake in Western diet.
