# Supplementary figures and images for: ANO4 (Anoctamin 4) Is a Novel Marker of Zona Glomerulosa That Regulates Stimulated Aldosterone Secretion
Source: Hypertension. 2019 Sep 30;74(5):1152–9. doi: 10.1161/HYPERTENSIONAHA.119.13287 (PMC6791498; doi:10.1161/HYPERTENSIONAHA.119.13287)

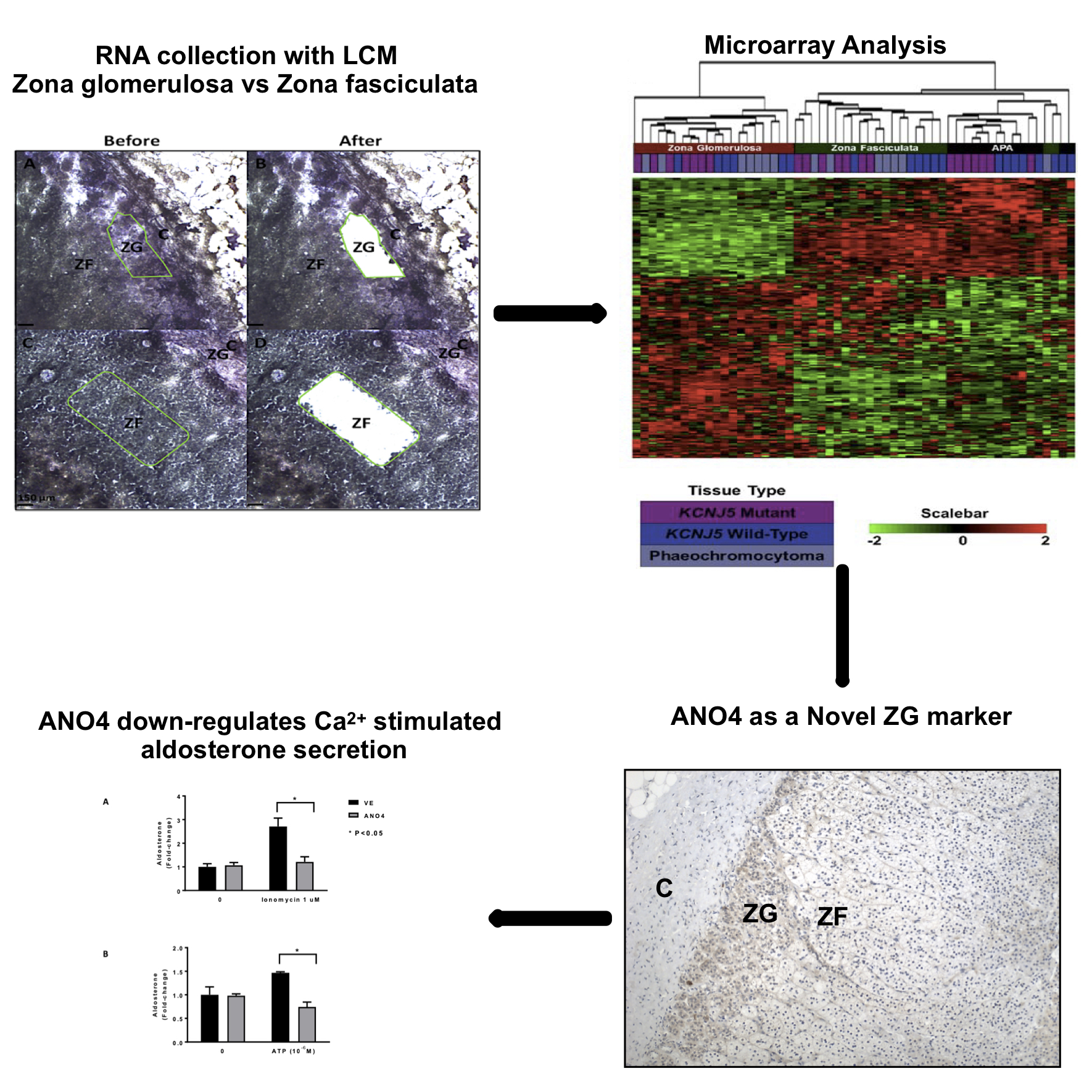

Supplement: Supplementary file 1 [file hyp-74-1152-s001.jpeg]
