# Supplementary material for: ANO4 (Anoctamin 4) Is a Novel Marker of Zona Glomerulosa That Regulates Stimulated Aldosterone Secretion
Source: Hypertension. 2019 Sep 30;74(5):1152–9. doi: 10.1161/HYPERTENSIONAHA.119.13287 (PMC6791498; doi:10.1161/HYPERTENSIONAHA.119.13287)
Supplement: Supplementary file 2 [file hyp-74-1152-s002.pdf]

Online Data Supplement

ANO4 is a Novel Marker of Zona Glomerulosa that Regulates Stimulated Aldosterone Secretion

Carmela Maniero[a], Paolo Scudieri[b], Lalarukh Haris Shaikh[a], Wanfeng Zhao[c], Mark Gurnell[d], Luis J.V. Galietta[b], Morris J Brown[a]

[a]The Centre for Clinical Pharmacology, William Harvey Research Institute, Barts and the London School of Medicine & Dentistry, Queen Mary University of London, United Kingdom Clinical Pharmacology Unit; [b]Telethon Institute of Genetics and Medicine (TIGEM), Pozzuoli, Italy; [c]Human Research Tissue Bank, Cambridge University, Hospitals NHS Foundation Trust, Addenbrooke's Hospital, Cambridge, United Kingdom; [d]Metabolic Research Laboratories-Wellcome Trust-MRC Institute of Metabolic Science.

Laser capture microdissection (LCM).

LCM was used to acquire samples of ZF, ZG and APA as previously described [21]. For differentiation of ZG from ZF, sections were stained with cresyl violet using the LCM Staining Kit (AM1935, Ambion, USA).

RNA extraction.

Samples were stored in RNAlater (Ambion, USA) and TRIzol® reagent (Life Technologies, USA) at -70°C till extracted for RNA. Total DNA-free RNA was isolated using the PureLink® RNA Mini Kit and DNase Set (Life Technologies, USA) according to manufacturer's instruction. Reverse transcription was performed using the Reverse Transcription System (Promega, USA) with a 1:1 mixture of random hexamer and oligo-dT primers according to manufacturer's instruction.

Microarray Assay

Microarray assay was performed using the Affymetrix Human Genome U133 Plus 2.0 Array by GenomicsCorelab, Cambridge. Fifty-six RNA samples acquired through laser capture microdissection were assayed—14 trios of ZF, ZG, and APA from patients with Conn's syndrome and a further 7 pairs of ZF and ZG adjacent to a pheochromocytoma. Seven of the APAs contained a somatic mutation in KCNJ5, whereas the remaining 7 were wild-type. Microarray results were validated by quantitative real-time polymerase chain reaction.

Data processing and analysis was performed using Affymetrix GeneChip Command Console Software and Partek GenomicSuite 6.5 (Partek Inc., St. Louis, MO). Gene expressions were portrayed as the summarized log-signal of the Robust Multichip Average (RMA) with quantile normalisation and median polish for probe set summarisation. One RNA sample from the ZG of ADR121 was not analysed, as it did not pass quality control. Genes were considered differentially expressed where the false discovery rate p-value was less than 0.05 and had a fold change (FC) >2. Validation by qPCR was performed on genes with FC >10.

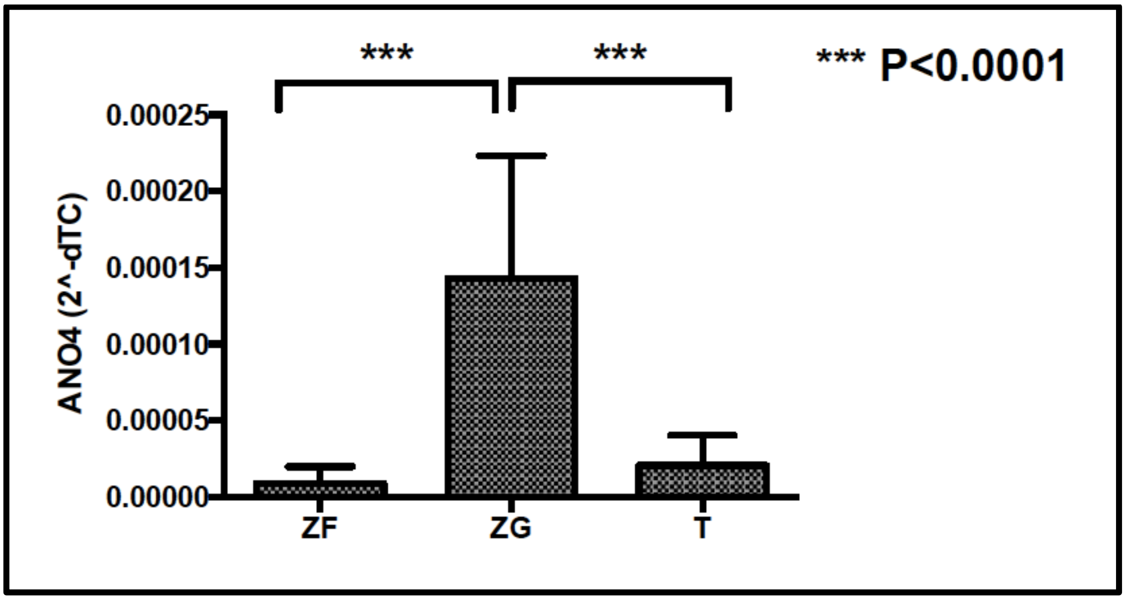

Figure S1. ANO4 qPCR validation of microarray findings performed on LCM samples from ZG, ZF and APAs of 7 patients with phaeocromocytoma, 7 patients with KCNJ5 mutant, and 7 patients with KCNJ5 wild-type APAs. Results are expressed as Mean and S.E.M of $2^{\wedge -dCT}$.

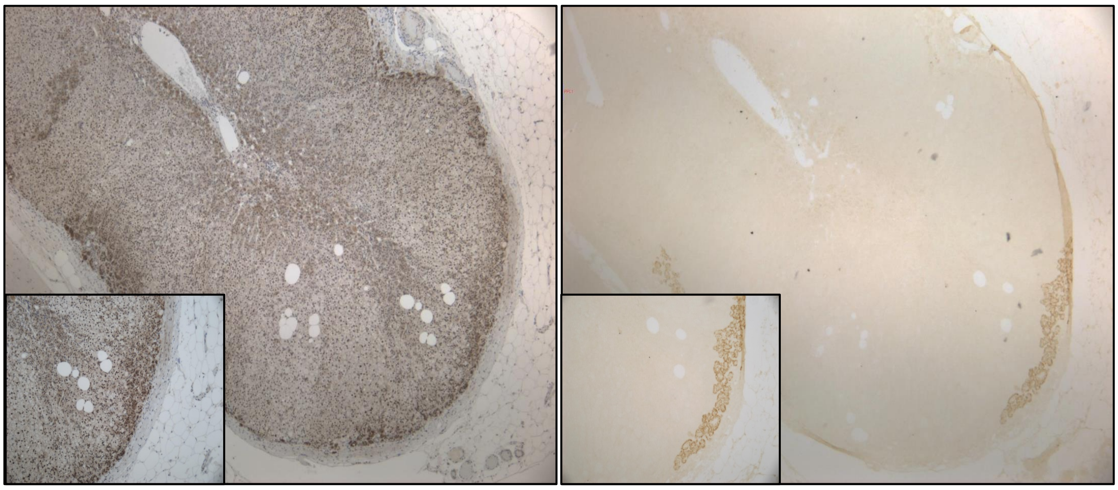

Figure S2: IHC of ANO4 and CYP11B2 in serial sections from adjacent normal cortex in a patient with phaeocromocytoma (4X and 10X magnification in the insert). Compared to ANO4, CYP11B2 staining is more patchy and localised in APCCs while ANO4 is expressed homogenously in the ZG and does not show any correlation with CYP11B2 expression.

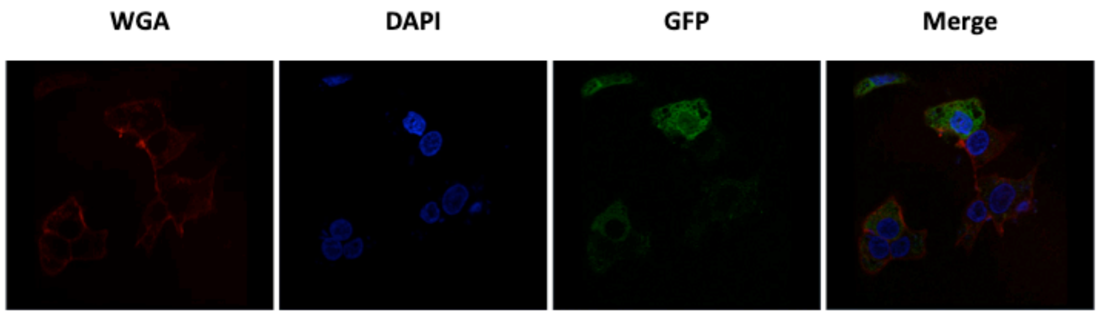

Figure S3: Immunofluorescence staining for transfected ANO4 in H295R cells. WGA (red) corresponds to cell membrane, GFP (green) corresponds to transfected ANO4, and 4′, 6-diamidino-2-phenylindole (DAPI, blue) stains nuclear DNA. GFP-tagged ANO4 appears to localize in the cytoplasm.

A

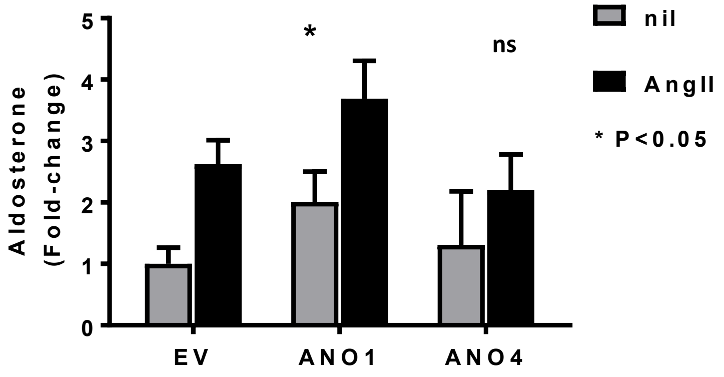

B

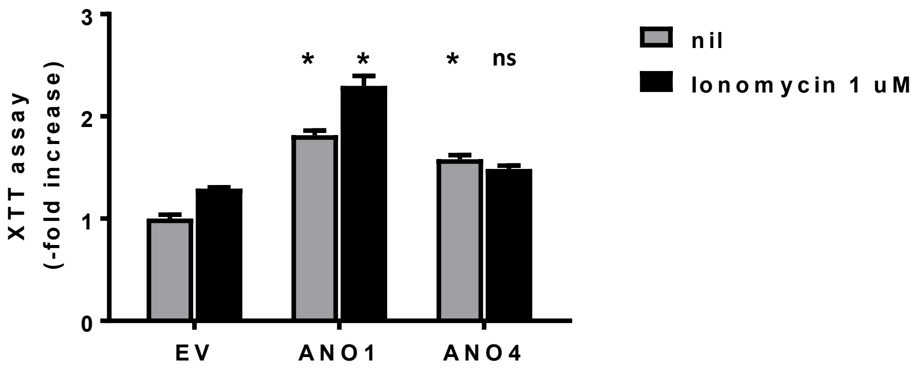

Figure S4: Effect of ANO4 and CaCC ANO1 overexpression in H295R cells on aldosterone secretion and cell proliferation in response to AngII and Ionomycin (1 µM), both increasing intracellular Ca2+ concentration.

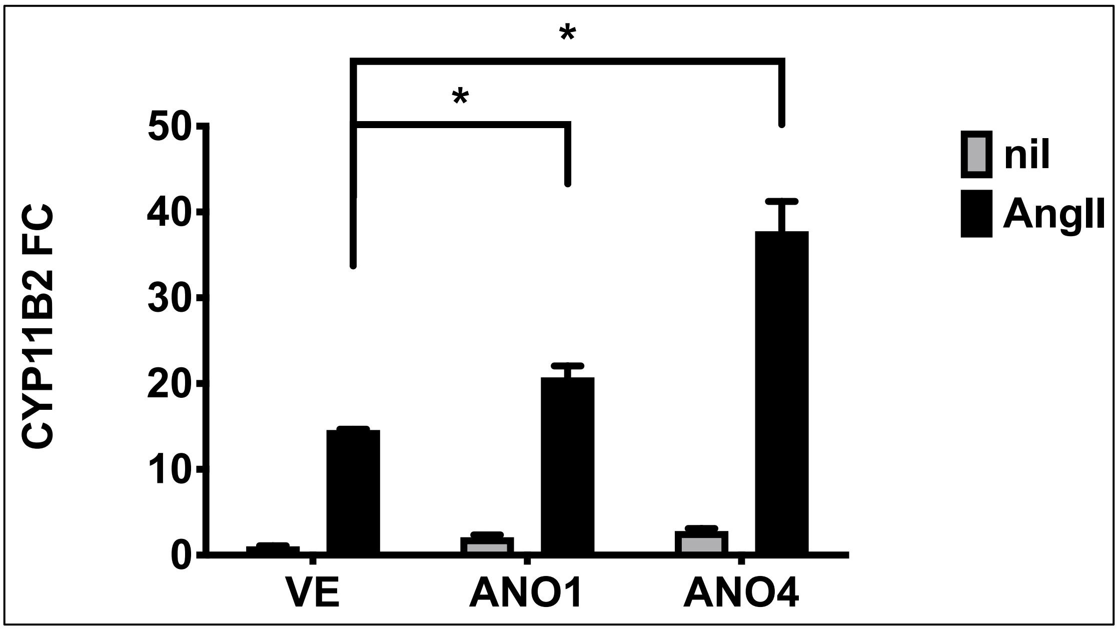

Figure S5: Effect of ANO1 and ANO4 overexpression in H295R cells on AngII stimulated CYP11B2 mRNA expression. CYP11B2 mRNA in basal conditions was 2, and 2.7-fold higher vs. controls in ANO1 and ANO4 respectively. CYP11B2 mRNA expression in response to AngII was 14.6, 20.7, 37.7- fold higher in comparison to basal controls in VE, ANO1 and ANO4 transfected cell, respectively. Ang II stimulated CYP11B2 mRNA expression was significantly higher in ANO1 and ANO4 vs. stimulated VE (* $P<0.05$).
